# Cognitive perspectives on maintaining physicians’ medical expertise: I. Reimagining Maintenance of Certification to promote lifelong learning

**DOI:** 10.1186/s41235-023-00496-9

**Published:** 2023-07-24

**Authors:** Benjamin M. Rottman, Zachary A. Caddick, Timothy J. Nokes-Malach, Scott H. Fraundorf

**Affiliations:** 1 Learning Research and Development Center, University of Pittsburgh, 3420 Forbes Ave., Pittsburgh, PA 15260 USA; 2 Department of Psychology, University of Pittsburgh, Pittsburgh, USA

**Keywords:** Longitudinal assessment, Medical boards, Feedback, Continuing medical education

## Abstract

Until recently, physicians in the USA who were board-certified in a specialty needed to take a summative test every 6–10 years. However, the 24 Member Boards of the American Board of Medical Specialties are in the process of switching toward much more frequent assessments, which we refer to as *longitudinal assessment*. The goal of longitudinal assessments is to provide formative feedback to physicians to help them learn content they do not know as well as serve an evaluation for board certification. We present five articles collectively covering the science behind this change, the likely outcomes, and some open questions. This initial article introduces the context behind this change. This article also discusses various forms of lifelong learning opportunities that can help physicians stay current, including longitudinal assessment, and the pros and cons of each.

## Significance statement

Medical Boards assess whether physicians have the knowledge and skills to practice safely and effectively and whether they are keeping up with current medical developments. Switching from using a summative test every 6–10 years to a longitudinal assessment will impact most of the nearly one million physicians in the USA and could have important consequences for the care they provide to the rest of the population. Thus, it is important to carefully consider the likely consequences of such a switch and to identify the factors that can make longitudinal assessment more successful. Furthermore, given that longitudinal assessment is one out of multiple lifelong learning opportunities for physicians, it is important to consider the holes that it might fill and gaps that remain. We review basic research from cognitive psychology as well as applied research that is relevant to answering these questions. More broadly, the idea of switching from high stakes summative tests to lower-stakes formative testing to provide learners with feedback so that they can effectively regulate their own learning is an important topic for all of education, including outside of medicine. Though this review of basic science, inspired by the switch taking place in medicine, is especially relevant to understanding lifelong learning, much of this research comes from shorter timescales, such as learning that takes place within a semester or academic year, and is therefore relevant to education more broadly.

## History of medical boards and goals of the project

Over the past several decades, all 24 Member Boards of the American Board of Medical Specialties (ABMS) began time-limited certificates and required physicians who were initially certified by the Boards, known as *Diplomates,* to take and pass an examination every 6–10 years to maintain their certification.^1^ Historically, these examinations took the form of point-in-time multiple-choice question assessments taken by Diplomates at secure testing centers, much like the examinations used for initial certification. These are best viewed as retrospective “assessments *of* learning” (i.e., summative assessments) designed to determine if the current knowledge base of a Diplomate remains at or above a level commensurate with certification in the associated specialty or subspecialty.

Certification assessments started to change in 2014 when the American Board of Anesthesiology began pilot work on their Maintenance of Certification in Anesthesiology (“MOCA Minute”) program (Sun et al., 2016). In contrast with traditional point-in-time examinations, MOCA Minute was designed as a proactive “assessment *for* learning” (i.e., a formative assessment) in which Diplomates completed a series of questions in one minute taken longitudinally over the course of the year. Participation was intended to assist Diplomates in keeping up with changes in medicine and to promote learning, retention, and application of knowledge in patient care. This approach draws on advances in cognitive psychology (Birnbaum et al., 2013; Brown et al., 2014; Cepeda et al., 2006; Dempster, 1988; Karpicke & Roediger, 2008; Roediger & Butler, 2011), including spaced learning and the testing effect, as well as upon recent advances in internet-based testing such as inclusion of hyperlinks to learning resources.

Physician certification programs have evolved in content, approach, and also in name over recent decades. Initial certification programs first required recertification before moving at the start of the new millennium into a new paradigm of Maintenance of Certification (MOC), emphasizing continuous professional development. Over the past decade, all 24 ABMS Member Boards agreed to develop and migrate toward continuing certification (CC) programs signaling that training and acquisition of medical practice knowledge and skill begin in medical school, are enhanced during residency, and are maintained throughout a specialist’s career.

Shortly after the introduction of MOCA Minute, other ABMS Member Boards began planning for more frequent, lower-stakes assessments as part of their assessment programs, and, as of mid-2020, all 24 Boards have announced programs that blend both “assessment of learning” and “assessment for learning.” In common across the programs are an emphasis on provision of specific immediate feedback on performance, timely identification of areas of strength and weakness (assessment for learning), use of aggregated performance over time to make summative decisions (assessment of learning) regarding continuing certification (Price et al., 2018), increased relevance of the assessments to Diplomates’ practice, and using an “open-book” format so that the questions focus more on reasoning rather than rote memory. At the same time, the programs are diverse in the frequency of the summative assessment, the participation requirement, the number of questions included, the time allotted per question, the use of spaced repetition, and the format of the aggregate feedback provided.

Because of the diversity of the longitudinal assessment programs across the specialty boards, the American Board of Internal Medicine along with the American Board of Family Medicine and the ABMS decided in 2020 to support research that reviewed the foundational science in cognitive and learning sciences and medical education underlying longitudinal assessment, synthesized the findings into recommendations for best practices, and identified key research gaps to be addressed. We—a team of cognitive psychologists from the University of Pittsburgh—were commissioned to do this work so as to present an unbiased view of the state of the research.

This research is intended to provide a theoretical framework for continuing assessment of physicians’ clinical knowledge. The framework presents a model of the foundational science, and it addresses some practical implications for the form that assessment and learning should take through a professional’s career, the frequency with which Diplomates should engage with continuing assessment, the potential of spaced repetition in the design of the assessment, the most appropriate ways to motivate learning, and the key areas of research that are important for helping the Board’s community to determine whether the longitudinal programs were in fact improving cognitive skills and, in turn, patient care.

We reviewed the foundational science behind longitudinal assessment and arrived at four critical themes: (1) cognitive skills must be kept current; otherwise, they will decline over time, (2) self-assessment is not always enough to reliably and effectively assess one’s own competencies or to guide one’s own learning, (3) testing enhances learning and retention of cognitive skills and knowledge, and (4) the role of motivation for learning in relation to assessments. These themes are presented in separate articles that accompany the current one.

In our research, we prioritized empirical findings from basic cognitive science and, where available, complementary medical evidence. Additionally, we identified gaps in knowledge and proposed a number of follow-up studies that would be relevant to longitudinal assessment of medical knowledge. Not at all empirical evidence is equal. To properly situate the strength of the evidence and claims made throughout this paper, we have attached evidence levels (EL) to in-text citations for empirical claims (Table 1). The evidence levels range from 1 to 6, with 1 being the strongest evidence (meta-analyses) and 6 being the weakest evidence (opinion papers).

**Table 1:** Evidence levels for in-text citations for empirical claims

Evidence level	Type of work
1	Quantitative meta-analysis
2	Narrative review
3	Multiple original experiments/randomized controlled trials (RCTs)
4	Single original experiment/RCT
5	Correlational or quasi-experimental study
6	Opinion paper

The rest of this article is organized as follows. First, we provide an overview of how the cognitive and learning sciences can inform longitudinal assessment. Second, we discuss some limitations to the work, in particular about how well basic research can be applied to lifelong learning in medicine. Third, we discuss the role of longitudinal assessment in comparison to other lifelong learning mechanisms, such as continuing medical education, clinical experience, and others.

## Cognitive perspectives on longitudinal assessment

**Fig. 1:**
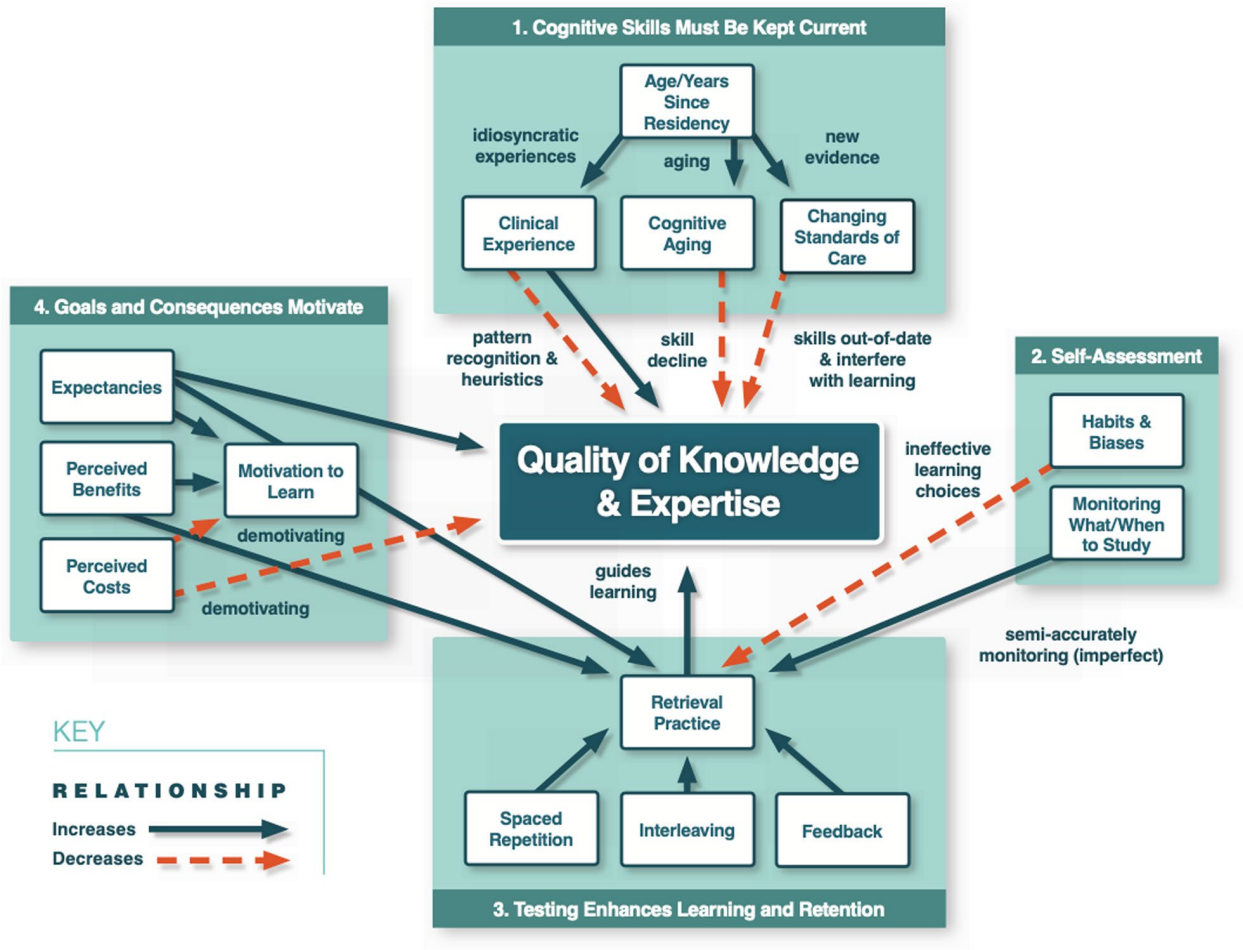
Synthesis of topics influencing quality of knowledge and expertise

Figure 1 presents our learning and assessment model of the role of longitudinal assessment in maintaining the quality of physicians’ knowledge and expertise. Arrows denote the causal processes or mechanisms that explain the relationships among the variables. Arrows with solid lines represent positive relations (relationships of increase) and arrows with dotted lines represent negative relations (relationships of decrease). The four boxes are used to place the variables into theoretical groupings discussed in each of our other articles. Below, we summarize and synthesize each of these theoretical groupings before we discuss the cross-cutting theme of feedback.

### Cognitive skills must be kept current

As physicians get farther and farther out of residency, three processes happen in parallel (Caddick et al., 2022). First, physicians accumulate more clinical experience over time. This extensive clinical experience can exert a positive effect on patient care—particularly in areas in which physicians choose to focus their practice—because it allows for quick pattern recognition, which often produces accurate diagnoses and other useful clinical decisions (Norman et al., 1989, EL: 5). However, it can also be negative insofar as a physician’s clinical experience is also inherently idiosyncratic, and some physicians choose to narrow their practices over time, which may leave gaps in knowledge and introduce bias by distorting perceptions of prevalence. These idiosyncrasies, biases, and gaps in knowledge can lead physicians to make incorrect diagnoses or decisions that deviate from standards of care (Choudhry et al., 2006, EL: 5).

Second, over time, physicians also experience cognitive aging. Research on cognitive aging suggests that, as physicians age, they will tend to rely more heavily on habitual routines, rather than learning new ones, and they may also have more difficulty balancing multiple tasks in working memory. The research we reviewed shows that, on average, older physicians do tend to provide poorer quality of care than younger physicians (Choudhry et al., 2005, EL: 2). However, the specific mechanisms of this finding are unclear because age is correlated with multiple other factors, including time since residency, changes in standards of care, the accumulation of (varied) clinical experiences and consequent changes in pattern recognition, and specialization or changes in clinical practice.

Third, physicians need to learn new standards of care as standards can change over time. Staying up-to-date can be difficult because it involves several processes (Cabana et al., 1999, EL: 2; Cochrane et al., 2007, EL: 2). In particular, physicians must (1) be initially exposed to a new standard of care relevant to their practice, (2) gain knowledge of the new standard, (3) agree with the new standard, (4) feel confident that they can implement it, and (5) remember to use the new standard when appropriate. Each individual barrier can be a challenge, and because there are multiple barriers, there are multiple potential points of failure to learn and implement new standards.

### Self-assessment is not enough

Is self-assessment enough to keep cognitive skills current? As we discuss in Fraundorf et al. (2022a), prior research does support the importance of accurately self-assessing one’s own skills and abilities (Metcalfe & Finn, 2008, EL: 3; Ohtani & Hisasaka, 2018, EL: 1; Tullis & Benjamin, 2011, EL: 5). Successful self-assessment includes at least two components. *Resolution* is the ability to identify one’s relative strengths and weaknesses, such as a physician’s areas of expertise (Eva & Regehr, 2011; Regehr et al., 1996). *Calibration* is the ability to evaluate one’s overall level of performance, such as whether a physician is overconfident, underconfident, or appropriately confident in their diagnostic and management decisions (Meyer et al., 2013; Podbregar et al., 2001; Zwaan & Hautz, 2019).

However, in self-assessment, individuals do not have direct access to either such component. Instead, they use “informed guesses” which, though somewhat accurate, suffer from systematic biases that are difficult to remove (Koriat, 1995, EL: 5; Koriat, 1997, EL: 5; Schwartz et al., 1997, EL: 2): For example, information that feels easy to process in the moment can lead individuals to overconfidence in their ability to remember it in the future (Kornell et al., 2011, EL: 3). People also tend to underestimate how much they will forget (Koriat et al., 2004, EL: 3). This implies that physicians may think that they need less continued training to maintain a given level of knowledge than they actually do; indeed, on the whole, physicians tend to be overconfident in their diagnoses (Berner & Graber, 2008, EL: 2).

Further, people tend to avoid many of the learning strategies that are best for long-term retention, such as self-testing, because the sense of difficulty they engender feels—in the moment—like poorer learning (Kirk-Johnson et al., 2019, EL: 3; Yan et al., 2016, EL: 3). Instead, people prefer other forms of learning that feel better, but are actually less effective. This implies that, if given the choice, many physicians will study in ways that are less effective or efficient than if directed by a longitudinal assessment program.

For these reasons, self-assessment must be supplemented by external sources of assessment, such as continuing certification programs, that can provide a more objective assessment of a physician’s knowledge and skills. At the same time, given that individuals do have some ability to accurately self-assess their own knowledge, this can potentially be leveraged by giving physicians some control over the topics included in the assessment.

### Testing enhances learning and retention

Whereas the goal of continuing certification programs has traditionally been to assess whether physicians are maintaining skills and keeping up with changing standards, the switch to longitudinal assessment presents the opportunity for testing to serve learning as well as assessment purposes. Although assessments are often viewed as merely tools for decision-making about one’s performance level, strong evidence (reviewed in Fraundorf et al., 2022b) indicates that being tested is a powerful learning experience in its own right: The act of retrieving targeted information from memory strengthens the ability to use it again in the future, so that new and old standards of care can remain distinct and readily accessible (Adesope et al., 2017, EL: 1; Rowland, 2014, EL: 1; Yang et al., 2021: EL 1).

Testing is further strengthened when followed by feedback (Rowland, 2014, EL: 1), a phenomenon too often lacking in medical practice itself, and by having tests spaced out over time (Cepeda et al., 2006, EL: 1; Phillips et al., 2019; EL: 2; Pyc & Rawson, 2009, EL: 3). Evidence indicates that greater frequency of testing yields deeper learning (Yang et al., 2021: EL 1). However, the optimal frequency and number of tests a physician takes should be weighed against the burden to physicians. Research suggests that topics that are hard to distinguish can generally be better learned by intermixing rather than presenting them one at a time (Brunmair & Richter, 2019, EL: 1), but there is a need for future research to identify the exact sequence that is optimal in medicine. Another benefit to creating a longitudinal assessment program may be that it results in physicians adopting more effective study and learning habits as they are guided to experience the learning benefits of self-testing (Ariel & Karpicke, 2017, EL: 4; Einstein et al., 2012, EL: 5; Shaw et al., 2011, EL: 3; Tullis et al., 2013, EL: 4).

### Goals and consequences motivate

Testing can also serve as an important motivator (Nokes-Malach et al., 2022). Physicians will be more motivated to study and practice their skills when the perceived benefits of doing so outweigh the perceived costs (Eccles & Wigfield, 2002, 2020; Wigfield & Eccles, 2000; Wigfield et al., 2016). The expectation of specific, challenging assessments can lead people to study longer and more meaningfully (McDaniel et al., 1994, EL: 3; Szpunar et al., 2007, EL: 4); thus, testing should be challenging enough to engender deeper and more effective learning but also not so difficult as to lead to expectations of failure (Bandura, 1997, EL: 2; Honicke & Broadbent, 2016, EL: 1; Pajares, 2008, EL: 2; Schunk & Pajares, 2002, EL: 2).

Physicians are also typically intrinsically motivated (i.e., internally driven) to learn and improve in their respective medical field. Emphasizing how maintenance of medical expertise aligns with physicians’ values can increase the perceived benefits of preparing for and engaging with longitudinal assessment to further facilitate one’s motivation to learn (Harackiewicz & Priniski, 2018, EL: 2; Schiefele et al., 1992, EL: 1). Longitudinal assessment programs would benefit from emphasizing congruence with the physicians’ interests (topics and scenarios; Walkington & Bernacki, 2018, EL: 2) and their educational and career goals (e.g., developing expertise and staying current), and by being established as an accurate measure of an important aspect of their knowledge and skills (Guo et al., 2016, EL: 5; Meyer et al., 2019; EL: 5; Putwain et al., 2019, EL: 5; Trautwein et al., 2012, EL: 5).

Decreasing or mitigating the perceived costs of the assessment is also important. More frequent, low-stakes testing may help reduce test anxiety and stereotype threat relative to less frequent, higher-stakes tests (Hinze & Rapp, 2014, EL: 3; Nguyen & Ryan, 2008, EL: 1; Shewach et al., 2019, EL: 1), which in turn can help improve study behaviors and test performance (Ackerman & Heggestad, 1997, EL: 1; Hembree, 1988, EL: 1; Sarason, 1980, EL: 2; von der Embse et al., 2018, EL: 1). Increasing a physician's motivation to learn, in turn, leads individuals to work harder, persist longer in the face of difficulty, adopt better learning strategies, and procrastinate less than when they are motivated by only external rewards (Hidi & Harackiewicz, 2000, EL: 2; Taylor et al., 2014, EL: 1).

### A cross-cutting theme: feedback on performance

One cross-cutting theme across this research is the role of feedback. In Caddick et al. (2022), we discuss how accurate and timely feedback is necessary for the development of expertise in any domain. However, the clinical systems provide imperfect feedback mechanisms. For example, if a physician makes an incorrect diagnosis, the patient may never receive the correct diagnosis, and even if they do, the correct diagnosis may not be conveyed back to the physician who made the incorrect diagnosis or instituted inappropriate treatment. Schiff (2008, EL: 6) reports that physicians often learn about their diagnostic success in an ad-hoc manner (e.g., malpractice subpoenas, running into a colleague) and that, as a result, physicians lack a reliable system for learning from past errors. In certain cases, feedback could be biased (e.g., a patient avoiding a physician because they were harmed by an error), and the low rates of autopsies in modern medicine have means that errors and misdiagnoses may never be discovered (Shojania et al., 2002, 2003).

In Fraundorf et al. (2022a), we discuss how, in the absence of external feedback, people need to rely on their own internal monitoring to assess what they do vs. do not know. Though individuals do have some ability to monitor what they do versus do not know, this internal monitoring is imperfect in a variety of ways. In particular, the poorest performers in a domain are the least accurate in their self-assessments, and they tend to overestimate their knowledge. This overestimation is believed to derive from the same lack of knowledge that caused them to perform poorly in the first place. Poor metacognitive accuracy is particularly problematic in high-stakes environments like medicine if a physician makes incorrect decisions with high confidence. Stepping back, it makes sense that insufficient feedback is the underlying cause of both poor knowledge/skills and subsequent overestimation of one’s knowledge. Therefore, we expect that better learning through testing with feedback should improve both accuracy and metacognitive understanding of one’s abilities.

We also discuss feedback extensively in our review of the testing effect (Fraundorf et al., 2022b). Though testing improves memory even without feedback of the correct answer, testing with feedback is even more effective. In that work, we also identified a number of open questions regarding precisely how and when to provide feedback.

In Nokes-Malach et al. (2022), we discuss how feedback is critical to several aspects of motivation. Feedback is one important factor in the development of beliefs of self-efficacy. Both positive and negative feedbacks influence one’s beliefs of self-efficacy. More generally, longitudinal assessments provide opportunities for individuals both to get multiple pieces of feedback over time and to improve self-efficacy with practice and sustained effort. Feedback is also critical to achievement goals and is needed to help one determine whether they are accomplishing one's goals. For example, to determine whether one is accomplishing a goal of self-improvement and increased knowledge, one needs feedback to compare performances over time. Feedback also plays a critical role in the impact of mindsets on performance and behavior. Growth mindsets have been hypothesized to be particularly important for situations where one receives negative feedback because mindset influences whether one persists in the face of setbacks. The type of feedback also matters. If one is given feedback, that highlights future opportunities for growth and improvement that feedback will be viewed differently than one-time, high-stakes, evaluative feedback. The latter often is viewed as a contributing factor leading to high test anxiety.

There has been growing discussion about the lack of feedback in medicine and different ways to begin to implement feedback loops to improve learning and safety (Cifra et al., 2021; Khazen & Schiff, 2021; McGinnis, 2013; National Academies of Sciences, Engineering, & Medicine, 2015; Rosner et al., 2022).

## Translating basic research to lifelong learning in medicine

We have endeavored to report what we view as the best and most relevant evidence out of a much larger body. Nevertheless, much of the research comes from basic science studies performed in psychology laboratories and a smaller set from more applied research in various settings, such as classrooms. An even smaller minority was conducted in the context of medicine, and some of these studies involve medical students or nurses in classroom settings rather than practicing clinicians. Thus, a vital question is how well this basic research applies to learning among expert physicians who have years of clinical practice.

This is a challenge in multiple dimensions. One dimension is simply that there are major demographic differences in that physicians are older. Though, in theory, this could make a difference, and though we cite evidence—for example—of age-related declines in working memory, we do not have specific reasons to believe that age-related changes interact with evidence such as retrieval practice or spacing. Another potential concern is the setting; perhaps laboratory and classroom settings are different from a standardized test setting. Again, we do not see theoretical reasons to be concerned that the setting would make a major difference. However, there are other dimensions that are potentially more concerning.

One issue is that continuing certification involves learning over decades—one’s entire working life—whereas almost all the studies cited, except for the few on continuing certification, involve much shorter time frames. Another concern has to do with the content. Though some of the studies do involve doctors reasoning about medical topics, many of the studies are about much simpler content that can be taught within the confines of a few hours, or at least within a semester. The raw amount of knowledge that physicians have, in terms of both breadth and depth, is orders of magnitude higher than that in many of these studies. Another concern, highly related to the previous points, is that most of this basic research was conducted not with experts but with novices, that is, people learning about material that does not tap into extensive knowledge systems that they have developed over many years. Despite these current limitations, we view these gaps in the literature as exciting opportunities to study basic science phenomena but in a setting of critical societal importance. For this reason, we believe that many of the studies we proposed would be of interest both to basic science researchers to advance theoretical understanding and to the ABMS Member Boards for their practical value.

## The role of longitudinal assessment in comparison with other lifelong learning mechanisms

The bulk of this paper so far has focused on the basic science of learning and the affordances of longitudinal assessment for learning. However, over the course of a physician’s career, they engage in multiple different forms of lifelong learning (see Wiese et al., 2022, for a review). All physicians continue to learn through continuing medical education (CME) and through personal experience with patients. Some physicians work in settings in which they receive best practice alerts and/or audit and feedback. None of these learning modalities is perfect, and all have strengths and weaknesses.

In this section, we attempt to characterize some of the most salient strengths and weaknesses of these different types of learning (summarized in Table 2). We first outline six features of learning opportunities that we consider to be important when considering how likely the opportunity would be to lead to learning. Then, we discuss six different learning opportunities for physicians post-residency and for each discuss the learning features that it has and does not have. We are not implying that each type of learning opportunity should have each feature; there very well could be benefits of having multiple different learning opportunities with different emphases. Rather, our goal is simply to create a framework for thinking about the similarities and differences between the learning opportunities.

**Table 2:** Comparison of features for keeping cognitive skills current across learning opportunities

Features of learning opportunities	Traditional 10-year assessment	Longitudinal assessment	CME	Clinical experience	Clinical decision support systems	Audit and feedback
Retrieval practice	Y	Y	N	Y	Y	Y
Feedback	N	Y	Y	S	Y	Y
Spaced	N	Y	S	Y	Y	Y
Self-directed	N	TBD	Y	Y	N	N
Consequences	Y	Y	S	Y	Y	Y
Authentic	N	N	N	Y	Y	Y

*Y* yes, *N* no, *S* somewhat, *TBD* to be determined

### Features of learning opportunities

We consider the following six features to be of critical importance for facilitating and tracking learning (though there may also be other features that we have not listed).

First, substantial work (reviewed in Fraundorf et al., 2022b, EL: 2) indicates that testing—*retrieval practice*—can be a powerful learning opportunity in its own right. Given that retrieval practice is so effective, we believe that it can be a critical component in lifelong learning.

Second, receiving *feedback* about one’s judgments is considered critical for becoming an expert (Kahneman & Klein, 2009, EL: 2), yet, in everyday practice, physicians often do not get useful feedback about whether their diagnoses and treatment plans are correct (Schiff, 2008; EL: 6). The important role of feedback is discussed in a cross-cutting section earlier in this article. A key point is that, even though testing is beneficial on its own, testing plus feedback is considerably more effective, especially for correcting errors. Nevertheless, the best way to structure feedback—particularly if a user answers incorrectly—merits more study. Ideally, feedback would promote learning and retention and thereby increase the likelihood the knowledge is applied in future patient care situations for which it is relevant.

Third, there is extensive evidence for the benefits of *spaced learning* (Fraundorf et al., 2022b): Learning is more effective and efficient when it is spaced out evenly across time than when it occurs in bunches (e.g., cramming right before a test). Given the robust evidence of the benefits of spaced learning, we added it as a desirable criterion here.

Fourth, another cross-cutting topic is the degree to which physicians should have control over both the topics that are included and the ways in which they engage in the assessment program. While *self-directed learning* may benefit physician’s intrinsic motivation, it is likely that having complete control over learning would lead to ineffective choices. In sum, the evidence suggests that learners should have *some* degree of control over the topics to be learned; they should not have complete control, nor should they have no control. The exact amounts and type of control are open questions.

Fifth, we have presented people are motivated by *consequences*—by perceived benefits and costs of taking a test and performing well on it (Nokes-Malach et al., 2022). For example, a certain level of arousal is beneficial for learning and performance, but too much is harmful. Furthermore, a sufficiently challenging assessment can facilitate both motivation to learn and ultimate performance, as long as the assessment is not perceived as too difficult. In sum, having some assessments with consequences is beneficial.

Finally, a topic that has not been discussed so far in this paper is whether learning is “authentic” and “naturalistic.” Within medical education specifically, and learning sciences more broadly (Barnett & Ceci, 2002, EL: 2; Chen & Klahr, 2008, EL: 2), there are concerns that if a learning environment is too artificial, it will do a poor job of preparing learners for the real-world tasks, and that if a *testing* environment is too artificial, it will do a poor job of predicting real-world performance. A theory from cognitive psychology called *transfer-appropriate processing * proposes that learning and retention are generally better when the learning environment matches the testing or practice environment (Blaxton, 1989, EL: 3).

Yet, others have observed that some efforts to create learning environments that are highly naturalistic—particularly high-fidelity patient simulators—do not produce learning benefits over low-fidelity simulators in skills such as auscultation, surgical motor skills, and critical care and crisis management skills (Norman et al., 2012, EL: 2). Others have found that scores on high-fidelity clinical simulations are too imprecise unless impractically large numbers of simulations are used and that multiple-choice questions can yield equally high criterion validity in a much shorter amount of time (Swanson et al., 1987, EL: 2).

The new situated cognition model of clinical reasoning takes the view of naturalistic or authentic reasoning a step farther. This model (Graber, 2020; Merkebu et al., 2020) stresses that clinical reasoning is not just in the head of the physician but is a much more complex process that involves interactions with the patient and medical team. Thus, advocates for the situated cognition model have suggested that assessments of clinical reasoning need to go beyond the simple cognitive decision-making that is assessed in multiple-choice tests and assess how the physician performs within the complex environment of a medical situation (2020b; Rencic et al., 2020a; Schuwirth et al., 2020; Torre et al., 2020). Doing so in a standardized way is obviously a major challenge and currently outside the scope of continuing certification program assessments. Still, the situated cognition model highlights the importance of authentic learning and assessment opportunities.

In Table 2, we classified each cell—whether a particular learning opportunity has a particular feature—as yes, no, or somewhat. However, for many of these cells the answers are more complex, and we discuss them below.

### Six lifelong learning opportunities

In this section, we discuss each of the six lifelong learning opportunities in Table 2 and, for each, address each of the six features of learning.

#### Traditional certification

Traditional certification examinations have a main goal of summative assessment, not learning in and of itself, although studying for the assessment should induce learning. Consequently, of the learning features reviewed above, the main one included in traditional assessment is consequences: If a physician fails and does not pass on repeated attempts within the time window, then they lose certification until they successfully pass. Though general feedback is provided about whether a physician passed the examination or not, as well as their percentile on the examination, and sometimes feedback on areas of weakness by topic, feedback specifically on individual items is not provided. This type of assessment can still serve as a form of retrieval practice—it could help reinforce knowledge that the physician already has—but because it does not include detailed feedback, it cannot help the physician understand their mistakes and could potentially reinforce their wrong answers.

Because certification examinations have traditionally been spaced far apart—10 years for many boards—instead of more frequent smaller examinations, they do not capitalize on the benefits of spaced learning. Although some traditional certification examinations allow some degree of customization, such as selecting content specific modules, generally most of the examination is standardized.

Lastly, because traditional certification examinations are largely multiple-choice examinations that take place outside of clinical practice, they are not as authentic as some of the other learning opportunities that more directly reflect—or are even embedded within—clinical practice, such as clinical decision support systems and audit and feedback.

#### Longitudinal assessment

Longitudinal assessment is designed to capitalize on certain learning opportunities that traditional assessments do not. In particular, whereas traditional assessment does not provide feedback about individual questions, longitudinal assessment does, which allows it to serve as a learning opportunity. Another change is that since the assessments happen more frequently, longitudinal assessment capitalizes on the advantages of spaced learning.

One topic that each board needs to consider is the extent to which learning will be self-directed, that is, whether physicians will get any choice in topics that they want to be assessed on and learn about. As argued above, we believe that giving physicians some degree of control could have advantages for motivation and for choosing topics that are most relevant to a physician’s practice. However, doing so also presents challenges for having a fair assessment of ability because physicians could game the system by choosing to be assessed primarily on their perceived strengths and not their weaknesses.

Proposals for longitudinal assessment do not change the consequences of failing from those of traditional assessments. However, longitudinal assessments will allow physicians to improve with each low-stakes assessment over the cycle at which there is a consequence (typically every 5 years). Longitudinal and traditional assessments are also the same with respect to authenticity in that both are fairly artificial and differ considerably from clinical practice (e.g., short verbal questions rather than the richness of actually interacting with patients).

#### Continuing medical education (CME)

There is a very extensive body of research on the efficacy of CME. Cervero and Gaines (2015, EL: 2) note 39 systematic reviews of evidence about CME over the period of 1977 until 2015, and they provide a helpful summary of this field. One overall conclusion is that, in general, CME tends to show small to medium effects on physician knowledge and performance.

A challenge in conceptualizing CME is understanding the range of activities that can sometimes count as CME. Group learning meetings (e.g., courses, conferences, lectures, workshops), online education, videos, reading journal articles or textbooks on one’s own, point-of-care learning (e.g., reading online references), and audit and feedback sometimes count as CME activities. For our present purposes, we focus on CME activities that are self-directed, such as choosing to attend a lecture or choosing to read an article on one’s own. One reason for this position is that most CME activities are in fact self-directed in that the physician can choose the topics to be studied—though, for example, a hospital system may require all medical staff to complete certain online coursework that counts as CME. Another reason is that it cleanly separates CME from other learning opportunities, such as audit and feedback; even though audit and feedback sometimes count as a CME activity, this is much less common, and audit and feedback have a very different profile in Table 2 than typical CME activities.

Another challenge for conceptualizing CME is how to view the use of point-of-care information services, such as looking up reference information to guide decision-making about an individual patient. Even though using online point-of-care references now often counts for CME, we include this as part of patient care since the context and goal is tied directly to decision-making for an individual patient; in contrast, most other CME, such as attending a lecture, is in a separate context outside of direct clinical care.

In sum, for our present purposes, we consider CME to take place outside of direct clinical care and to be self-directed in that the physician chooses the topics they want to learn about, though these are not always true of activities that count for CME credit.

Unlike all of the other learning opportunities in Table 2, CME activities usually do not involve retrieval practice. For example, in a didactic lecture, or when reading an article, the majority of content is simply presented without testing the learner first and then providing feedback. Of course, sometimes presenters may choose to ask the audience questions, but even if this is done, it usually comprises a fairly small amount of the total content being covered. Correct information is conveyed to the learner, so even though it is not in the form of feedback after being tested, the learner is still exposed to answers about the content.

We describe CME in Table 2 as “somewhat” providing spaced learning. Physicians can choose when to engage in CME activities, so it is possible that they complete many CME activities close to the deadline. On the other hand, given the large numbers of hours of CME requirements, presumably they are often completed in bits over longer stretches of time.

As explained above, our definition of CME for the purposes of this report is that it is self-directed, though in reality there are sometimes CME activities that are not self-directed. We rate CME as “somewhat” self-directed in Table 2 because many states require CME to remain licensed, and being licensed is a requirement for many jobs and board certification. However, many CME activities do not test knowledge and simply record that the activity was completed. Therefore, the consequences are tied to the minimal standards for completion, not tied to success. Lastly, most CME is not authentic in that learning takes place outside of clinical care.

#### Clinical experience

Physicians’ daily experiences with patients, and any accompanying efforts to search for information to guide decision-making about the patient, can serve as a valuable opportunity in many respects. Each patient encounter serves as a retrieval practice experience because a physician retrieves knowledge and practices skills. And because a physician has many experiences with patients, it is clearly spaced out over time. Furthermore, clinical practice is clearly an authentic experience.

However, there are some other features of personal experience that make it a suboptimal learning opportunity. First, as we have already discussed above, the feedback from personal experience is imperfect. Sometimes a mistake will become apparent later, but often a physician will not know about mistakes that they made.

Second, physicians face many different sorts of consequences in daily practice. The most prevalent consequence are patients’ health outcomes. Since physicians are motivated to help patients achieve their health goals, medical errors are associated with a number of subsequent psychological consequences for physicians, such as a decrease in quality of life, burnout, and depression (West et al., 2006, EL: 5). Other consequences can include legal action for malpractice. However, since many mistakes are not discovered and therefore there are no consequences, we rate clinical experience in Table 2 as only “somewhat” yielding consequences. Furthermore, the *Improving Diagnosis in Health Care* report (National Academies of Sciences, Engineering, & Medicine, 2015, EL: 6) suggests that guilt, shame, and legal action are likely not productive consequences for learning (see also avoidance-based goals; Nokes-Malach et al., 2022). Instead, this report recommended adopting a non-punitive culture and finding ways to close the feedback loop so that errors are more frequently and quickly discovered.

Lastly, in Table 2, we label clinical experience as self-directed. For each individual patient, the physician decides whether to make a clinical decision immediately or whether to look up information in online resources or consult with colleagues (Burden et al., 2013; Cook et al., 2014; Ely et al., 2005; Moja & Kwag, 2015); such decisions are self-directed. The best evidence suggests that higher rates of use of electronic knowledge resources are associated with better knowledge and patient care (Maggio et al., 2019, EL: 1). Still, physicians make the choice of when to look up information, and they often do not seek answers to questions that they have (Ely et al., 1999, EL: 5). Perhaps seeking out answers more frequently could make daily clinical experience more effective as a lifelong learning activity, though of course physicians have limited time in daily encounters to do so.

#### Clinical decision support systems

*Clinical decision support* (CDS) systems, otherwise known as *best practice alerts* (BPAs), *electronic health record alerts*, or *clinical reminder alerts*, are systems built into the electronic medical record that provide health providers with recommendations and alerts about patient care (e.g., Berner, 2007, 2009; Middleton et al., 2016; Musen et al., 2014). Among others, they include reminders that a patient should get a flu shot, prescription alerts about drug-drug interactions, alerts that a patient is starting to deteriorate, and suggestions about potential diagnoses. Despite the prevalence and diversity of CDS, the total number of high-quality studies eligible to be reviewed in meta-analyses are still fairly modest, and researchers have not specified why some alerts work better than others (Moja et al., 2014; Shojania et al., 2009, 2010). Due to the ubiquity of CDS generated alerts, there are calls to make alerts and reminders more relevant to avoid alert fatigue (e.g., Embi & Leonard, 2012; Hussain et al., 2019; Kesselheim et al., 2011; Phansalkar et al., 2013).

Despite the challenges of alert fatigue, CDS have the potential to benefit clinicians for several reasons (Chen et al., 2019, EL: 6; Middleton et al., 2016, EL: 6). First, CDS and other forms of technology can help separate tasks that can be done by others in the medical team from those that need to be done by the physician (e.g., Sinsky & Panzer, 2022). For instance, physicians often experience *cognitive load*: Decision-making taxes and sometimes overwhelms the limited capacity of humans to hold information in mind and use it, a capacity that can be further reduced by stress, emotion, and uncertainty (Szulewski et al., 2021; EL: 2). CDS can reduce such load by allowing physicians to *offload* some tasks—that is, to leave them to an external source like the CDS rather than one’s own mind (Risko & Gilbert, 2016; EL: 2). Second, CDS may lead to improved patient care even when the presented alert is not learned or remembered by the physician (e.g., a system may recommend the right antibiotic to prescribe, which is beneficial even if the physician does not remember this in the future); indeed, this is the often seen as the primary intended benefit of CDS.

A third possibility, of particular interest given our focus on learning, is that CDS alerts may provide valuable learning opportunities for physicians across many dimensions. Some of these dimensions are directly tied to the fact that they are part of the clinical experience.

CDS systems involve retrieval practice with feedback. Consider a physician prescribing a medicine and receiving an alert about a potential drug–drug interaction. This can be viewed as a type of retrieval practice in the sense that, when entering the prescription, a physician tests their knowledge of whether it is appropriate for this given patient and their other prescriptions. If the alert raises an important drug-drug interaction that the physician did not remember or consider, this could be a useful learning opportunity. Or, they may have already considered this interaction but decided to prescribe it anyways, in which case it still is reinforcing correct knowledge.

CDS alerts are spaced in the sense that they occur frequently during patient care, and authentic in that they are embedded in patient care. And, they are not self-directed in that physicians usually cannot turn them on or off. CDS alerts typically do not have any consequences attached to them, aside from the consequence of the patient’s health outcomes intrinsic to clinical practice.

One weakness with CDS systems in terms of providing learning opportunities is that the feedback that they provide is often imperfect. Physicians often override alerts and ignore or reject the suggestion—often for good reasons, such as the alert being generated by incomplete or incorrect patient data, logic that does not perfectly fit the patient, or others (van der Sijs et al., 2006, EL: 6; Middleton et al., 2016, EL: 6). Thus, for the foreseeable future, CDS systems can only be viewed as suggestions and imperfect feedback rather than authoritative feedback as would occur in longitudinal assessment or CME. Thus, in Table 2, we list as only “somewhat” present in CDS systems. Still, it is likely that this sort of feedback can be useful as a learning opportunity (Goodnough et al., 2014, EL: 5; Chen et al., 2015, EL: 5). Indeed, in a large-cluster randomized study that evaluated the addition of CDS reminders on top of audit and feedback relative to audit and feedback alone, physicians who received the point-of-care reminders were more likely to do the recommended task (e.g., prescribe a drug or vaccine, order a test, perform a screening, encourage smoking cessation) for all 10 clinical conditions tested, suggesting that CDS systems can be an especially effective form of feedback (Coma et al., 2019, EL: 4).

#### Audit and feedback

*Audit and feedback* is a quality improvement technique in which an individual’s performance is measured and compared to a desired professional standard, and then, the individual is given feedback about their performance. Though initially done in more cumbersome and time-consuming ways, there are newer automated systems (Tsang et al., 2022). Two meta-analyses found that audit and feedback tends to produce small but often statistically reliable improvements in meeting professional standards (Hysong, 2009, EL: 1; Ivers et al., 2012, EL: 1). The improvement seems to be larger for healthcare professionals starting out at lower levels of performance and when specific suggestions for improvement are provided (Hysong, 2009, EL: 1; Ivers et al., 2012, EL: 1). However, most research on audit and feedback does not explain why audit and feedback sometimes works better than other times, nor how to design the best audit and feedback systems for particular situations (Gardner et al., 2010; Grimshaw et al., 2019; Ivers et al., 2014). One suggestion is that providing timely feedback on specific actions is likely to be the most helpful (Tsang et al., 2022).

With regard to our dimensions in Table 2, audit and feedback has a very similar profile compared to CDS, though with some differences, because both are built on top of clinical experience.

Audit and feedback involves retrieval practice in the sense that physicians test their knowledge and skills daily in clinical work. Feedback is a core component of audit and feedback; one difference compared to CDS reminders and alerts is that the feedback is delayed and grouped together (e.g., given every month) rather than at the point of service. Learning is spaced over time naturally in clinical practice. Learning is not self-directed in that it is usually the organization, not the individual physician, that decides to implement an audit and feedback program, and usually there is not a way to opt out. For consequences, similar to CDS, audit and feedback typically does not have any consequences aside from the patient’s health outcomes, which is intrinsic to clinical practice. A few studies have investigated the role of adding financial incentives on top of audit and feedback, with mixed results (Ivers et al., 2012, EL: 4).

In sum, audit and feedback is similar to CDS alerts on the dimensions that we covered. Both have many desirable features of learning opportunities, though both require additional research into how to make them most clinically effective and least disruptive. Analyzing them from a perspective of how they promote long-term learning, retention, and behavior change could be helpful in this regard.

#### Summary

Our goal with Table 2 is not to classify certain learning opportunities as better or worse, but to show how they are different in terms of important dimensions of learning and therefore have different strengths and weaknesses. For example, though there are a number of weaknesses with CME in terms of learning, a strength is that it allows for a very high degree of self-directed learning. A physician who has identified an area of weakness may be able to devote a lot of time to learning that topic. Collectively, these varied learning opportunities fill different sorts of knowledge gaps. That said, it seems to us that longitudinal assessment fills a similar role as traditional certification in that they both provide retrieval practice and consequences, but longitudinal assessment provides a superior learning opportunity through its use of feedback, spaced learning, and potentially being somewhat self-directed.

## Conclusions

We report results from a project evaluating a wide breadth of research related to the development and maintenance of expertise in physicians. We provided evidence for four major themes regarding physician expert performance: (1) cognitive skills need to be kept current, (2) self-assessment is not enough, (3) testing enhances learning and retention, and (4) goals and consequences motivate. We created a learning model detailing our understanding of how these complementary themes interact and how they contribute to a physician’s knowledge and expertise as related to patient care. Lastly, we discussed whether other lifelong learning opportunities for physicians meet various psychological considerations that are believed to benefit learning. Going forward, there is considerable potential for the cognitive and learning sciences to collaborate with medical boards to conduct studies of longitudinal assessment programs that both test ways to improve learning within longitudinal assessment and advance the basic science of learning.

## Data Availability

Not applicable.

## Footnotes

1 Most Boards had additional requirements for maintenance of certification, including possession of an active, unrestricted medical license, acquisition of a specified number of continuing medical education credits, and engagement in quality improvement projects.
